# Supplementary material for: Transgenic expression of Nix converts genetic females into males and allows automated sex sorting in Aedes albopictus
Source: Commun Biol. 2022 Mar 7;5:210. doi: 10.1038/s42003-022-03165-7 (PMC8901906; doi:10.1038/s42003-022-03165-7)
Supplement: Supplementary file 2 — Supplementary Information [file 42003_2022_3165_MOESM2_ESM.pdf]

Supplementary Information for
Transgenic expression of *Nix* converts genetic females into males and allows automated sex sorting in *Aedes albopictus*

Célia Lutrat, Roenick P. Olmo, Thierry Baldet, Jérémy Bouyer and Eric Marois

Correspondence:
Célia Lutrat
Email: celia.lutrat@outlook.com
Eric Marois
Email: e.marois@unistra.fr

This PDF file includes:

Supplementary Notes 1 and 2
Supplementary Figures 1 to 4
Supplementary Tables 1 to 6

Other supplementary materials for this manuscript include the following:

Supplementary Data 1 (separate file): Model output from all statistical analyses and performance assessment

Supplementary Data 2 (separate file): Wing length measurements (Fig. 3b)

Supplementary Data 3 (separate file): qPCR data (Fig. 4)

Supplementary Data 4 (separate file): COPAS data for SM9 line (Fig. 6a)

Supplementary Data 5 (separate file): COPAS data for 1.2G line (Fig. 6b)

Supplementary Data 6 (separate file): COPAS data for 3.1G line (Fig. 6c)

Supplementary Note 1: Sequence of the *Ae. albopictus* genomic region amplified for cloning *Nix* promoter.

In the sequence below, *Nix* promoter sequence is included in the 1,916 bp sequence highlighted in grey, the 5' UTR sequence of the *Nix* gene is not highlighted, while the ATG start codon is highlighted in blue.

TCGCATTTTATGAGTAAAGGCCCATTACTCATATATGGGTAAAGTGCTTTTTG
TAAAAAATGAGTAAATCGATTTACTCATAAATTATAATTTCACCGTGTTTACT
TTTCGTCTTTTGTTATATTTTTGAACTGTTAACTATTTTGTATTTATCATTTAAT
TCATACAAAATTTTGCGTTTATTTTGTAGTTTATTGCAATCTACGAAAATTATT
AATGAATTTTTACATGTTTCAAATACTCTTAATCATTTTCCTCCTGGAAAACA
GACCTTCCGAGACGTAGCTCTTTGAAGTTTTCATAATGATCCATGTATGAAAT
TTTCCCCTCCGGCTGTTGTTCATCGCGCAGTTGTTCCTGTTCTTCCGTTCCTCG
TTCTTCCGGCGCTTGAAGCCTTGGTGCAGTCGGAACTGTGGAAAAGACTGAT
ATATTTTTATTTCACGGTCGTTAATGAAAATGATGCTGTCACTATGGAACTTA
CCTGCAAATTCCATGCTTTTGTAGCCTTTCTTGTAGCTCCACATGATGATTTGT
TGCTCCTGTATTATCTTCGAGTACCAAACCTTGTGAAACGGAAATAATCCATC
GTAAATGAAAATTGAACGGTATCAATTTATCCAGGAAGTACTTACCGATTAA
AATATCAATGTATTTGGGAACCTCGACTCTAATCCGCGTGGGAATCCGATGG
ACGCGGTATCGGTCATTGAACCCGATGCTGAAGGGCCTCGACTGGCTATAGC
GGTGCCGTGAAAATCAAAATATCTAAAATTTCCCGATAGTGTTATGATCAGG
GGATCCGCATGCGGCAAGGTAGGCCTACTGCTCATCTTTTGCGAGTGTGTTAG
TGTGACTGGCAACGCGACCAGAAGTCTAGTCGAATCCTGCTTGGAAATCGGT
TTGTTTTGTCGATGGGGGTGGCCCGCGAAATCGCATTGTGTTGTTTGGGAAGG
CCCGCTCGATGCATGTTAGGTAGGCGTAGTTTGTTCGAGTTTGACGTGCCTGC
ACCCTCCCTGGTTATGATGGCGCACTAATGCATTCCGAGAAAAGTTGCTAACT
TGAAAATTTTCATCCAAATATGGGTAAGTTCATTTACCCATGTTTGGGTATAG
CGATTTTACCTATAATATGGGCAAATTTGACCGTTTTTCAAAGGTATATTTTA
CCCATATTATGGGTAATGCATATGAAACCCAAATATGGGTAAATCAACTACT
GATCTATGGGTAAACTGATCTTAGCGTGTATGTTTCACACGTGACATTCACTG
GTTTTACAATTGGCTTCTTTAGCAGTGTTGGGATAATTCCATATTATGAATCT
GCATGCCAAACTGGGCCGAAATCCAAATTTTCATCAATTTTGGTGCACGGGA
ACCTATTTAAATATCAATTTGAAGTTTGTATGGGAGCGATTTGTCGAATCACC
CCTCGTTGCATTTTGTACTGGGCGGAGCTGTCAAACAGTTGCCTAGCTGTCAA
AAGGTGATTTCGAATAATCTCTTTGAAATTGATTTTAGGTATCAAAATAAAGT
TCTAAAAATCTGAAAAAAATCATAGTGGCTCAGAAAAAGGTGCTCTTTCGTA
TAAAATCAAAAAATGAACACTTTTTTCAAAATTTAAAAACCCAATTTTCGCAA
ATCTATAGGGCTCTGCACGAAGTTCTCTCCCTCTCTTTCGCTCTCATTGAGATT
TTGTAAACAACAAGGCCAGGAAATGTCAAAATCCCATACAAAATCAAAACA
GTGCAGTGCCCTATATGTAAAACACATCACTCCGACGTGTAAATTTTTTTGAG
TGTTGATTTAATCAAAGTGAATAAAAATATTAGTTTTATGACATACTTGTTTT
CTGAGTGTAGCAAAAATATGAAAACACATTTTTGTACTTTGAATGTTAAGCGT
GTATGCTTTTTGTGTCAATATGTCAATTGTTAAACCCATGTTAATAGTTTTAAT
TTTTTTTTAATCAAATTCTTTTTTTAAGTAATG

Supplementary Note 2: Male-specific amplification pattern of one of the copies of the *myo-sex* orthologue.

PCR amplification was performed using primers Myosex369-F and Myosex369-R targeting the end of the first intron of *myo-sex*. The expected product with this primer pair was 997 bp as provided below. The discovered male-specific product carries a 664 bp deletion (highlighted in grey) and is present in genetic males only.

AGGCCATACTAACCTTCCGTAAATCACGCGGTTGGCCACTACCGTCAGCACC
ACGATAATGCTGAGTACAAAATTTAGATTTCTTCAACGTGTTGTACAAAATAC
AAAAGCGCGATCGCTCGAGGGCTAAGAATTAATGAAGAAAATTGCAAATCA
ATTACATATTTATACAAAATCATAACAATAGGTATAGATGTTTGCAAAGATGT
TCGTTGACAGCTTGAATCTCGAAACAGAGATGAATAGATTGAACTATAAAAC
AAACTCGATCAAAAATAACACAGCGTAACAAAAATAACTTTTTGTATGTCTC
TAGAGCAAACTTACGTGTCTCCGAAGGATTTTGGGCCGCTGAATCCGAATCT
GGGCTCAGATTTGCTCTAACACGTCACAATTTTGAGCTATACCTCAATTTATA
GGGCAAAATATGCGATTTTGGGCTTTTTTGACTGCAAGCCATTAAGCAAGGA
AATATTTTTTTAAGCAATCAAAAGGTTAATTGGTCAATTAACATCTAAATTAC
GACTCATGCAAAATATTTCGTTTTACCAAATCGAATTTGATAGTTTTAAGCGA
TTTATGTTAGGTACGATATTTCCCATATAAGTCAGCCTCCAAAAGTTGCATGC
AAGTTTTCATGCTAACATAAAATGCTTAAATCCATCAAATTTGATTAGGTAAA
ACGAAATATTTTGCATGAGTCGTTAATTTAGATGTTAGTTGACCAATTAACCT
TTTGATTGCTTAAAAAAAATATTTCCTTGCTTAATGGCTTGCAGTCAAAAAAG
CCCAAAATCGCATATTTTGCCCTATAAATTGAGGTATAGCTCAAAATTGTGAC
GTGTTAGAGCAAATCTAAGCCCGGATTCGGATTCAGCGGCCCAAAATCCTTC
GGAGACACATAAGTTTGCTCTTGAGACAAAAAAAATGTTGCGCTGTGTAATC
TGTTAAAAACATTGTCCTACATTTTTTGCGCTCCATTGTTACTTCATTGTAT

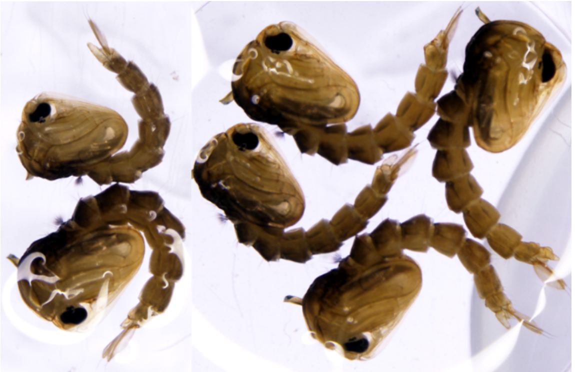

Supplementary Figure 1: Intersex phenotypes in SM9 line prior to purification. The two pupae on the left side of the picture are a control male and a control female from SM9 line. All four pupae on the right are representative GFP-expressing intersex individuals with deformed genitalia found in the SM9 line prior to elimination of additional non-fully masculinizing transgene insertions. Pictures were taken under a binocular microscope with white light.

a

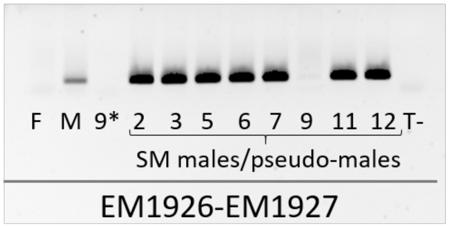

b

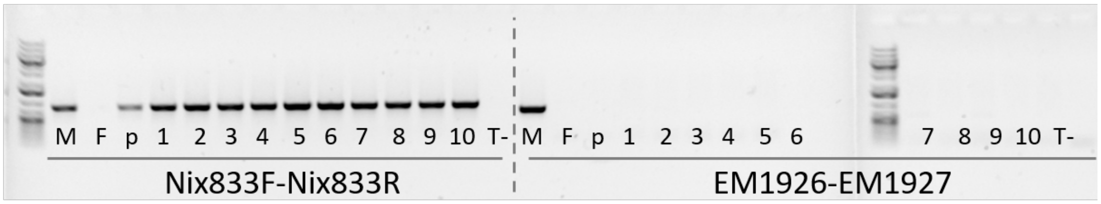

Supplementary Figure 2: Detection of lines composed exclusively of genetic females in lines carrying the *Nix*-OpIE2-GFP plasmid. a) PCR amplification of genomic DNA from pooled phenotypic males using primers EM1926-EM1927. This primer pair is located within the second intron of *Nix*, absent from our construct, and thus amplifies only DNA from genetic males. Genomic DNA was extracted from pooled pupae and analysed in the following order: WT females ("F", negative control), WT males ("M", positive control), SM9 partially masculinized female ("9*"), males or pseudo-males from selected lines arising from injection of the first OpIE2-GFP -marked *Nix* expressing plasmid (numbers 2 to 12), negative control without DNA template ("T-"). This PCR allowed identification of line SM9 as composed exclusively of genetic females. **b)** Screening of 10 SM9 individual males (labelled 1 to 10) with primer pair Nix-833F-Nix-833R amplifying any *Nix*-bearing genomic DNA [1] and primer pair EM1926-EM1927 for endogenous *Nix* only. The following controls were used: "M" is WT male positive control, "F" is WT female negative control, "p" is the pooled SM9 male DNA from top panel, and "T-" is a negative control without template DNA. This PCR confirmed that all phenotypic males in the SM9 line were pseudo-males. PCR products were resolved on a 0.8% agarose gel with 0.2µg/mL ethidium bromide.

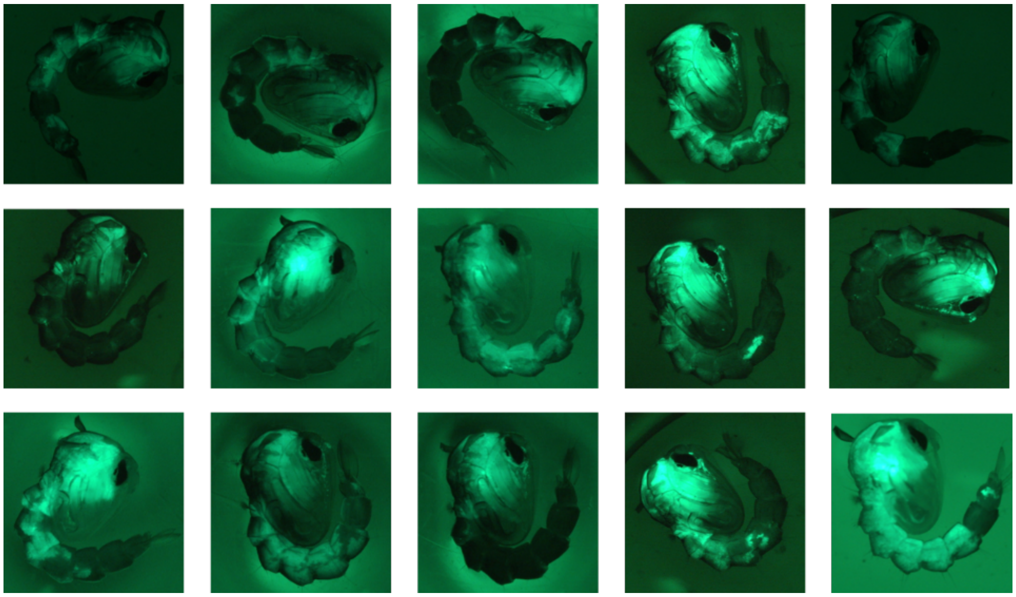

Supplementary Figure 3: Gallery of male SM9 pupae arising from CRE-injected embryos, showing local demasculinization in the posterior pole. All adults that emerged from these pupae had male heads and female genitalia. Pictures were taken under a binocular fluorescence microscope using a GFP filter.

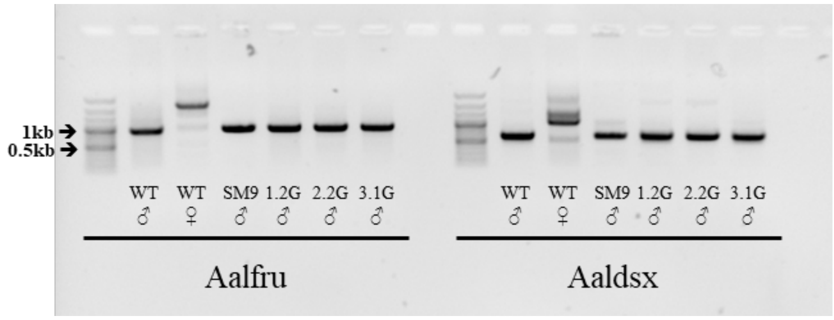

Supplementary Figure 4: Sex-specific *fruitless* and *doublesex* splicing patterns. mRNAs from WT male, WT female, SM9 pseudo-male, 1.2G pseudo-male, 2.2G pseudo-male, and 3.1G pseudo-male pupae were extracted, reverse-transcribed into cDNA, which was used for PCR. Products were resolved on a 1.5% agarose gel with 0.2µg/mL ethidium bromide. Left side: RT-PCR with *fruitless* sex-specific primers for which the male product is expected at 987 bp and the female product at 2010 bp. Right side: RT-PCR with *doublesex* primers, for which the male product is expected at 620 bp and the female product at 1062 bp.

Supplementary Table 1: Sequences of primers used in this study.

Name	5' – 3' sequence	Use
**EM1926**	CCCTCAATTTTCCGCCAACTATT	655bp amplicon in intron 2 for detection of endogenous *Nix*
**EM1927**	AATCTTTGGTGCGCCGTGTC	
**Myosex369-F**	AGGCCATACTAACCTTCCGT	Genomic amplification of a non-coding part of the *myo-sex* copy from scaffold 369 (end of intron 1).
**Myosex396-R**	ATACAATGAAGTAACAATGGAGCG	
**EM2145**	CAAGTTGGTGACGATCCCGA	For RT-qPCR of mRNA from *myo-sex* orthologues
**EM2146**	GTTGGGTAGAGCAACGGTGA	
**EM2147**	CGCCGGAAAAACGTATCCACT	For RT-PCR of mRNA from LOC115254984, a candidate *myo-fem* orthologue
**EM2148**	GCTGGTTCCAGGTTAGTTGG	
**EM2149**	CCCGTGCTGAAGAGTTGGAG	For RT-PCR of mRNA from LOC115254986, a candidate *myo-fem* orthologue
**EM2150**	GTGGACAGACGTTGCTTAGT	
**EM2151**	GTAGGCATCTACGAGCCCAA	For RT-PCR of mRNA from LOC109402113, a candidate *myo-fem* orthologue
**EM2152**	CCAACCTGTACCACTGGCTT	
**EM2153**	CATTGGAAACATTCCCGCCG	For RT-PCR or RT-qPCR of mRNA from *Nix*
**EM2154**	ACTGCCGGTTTCACATCACA	
**EM2170**	ACGTGCCGAAGAATTGGAAG	For RT-qPCR of mRNA from LOC109402113, putative *myo-fem* orthologue
**EM2171**	TTCTAAGGCAACACACTTCTGA	
**EM2174**	CGTGCCACCCTTCTTGGTAA	For RT-qPCR of mRNA from LOC115254984, candidate *myo-fem* orthologue
**EM2175**	CCTCCAACTCTTCTGCACGG	

Supplementary Table 2: Sex ratios of transgenic versus WT lines

Line	Replicate	Males	Females
**WT**	1	125	116
**WT**	2	128	128
**WT**	3	74	67
**SM9**	1	936	745
**SM9**	2	677	590
**SM9**	3	430	308
**1.2G**	1	889	1027
**1.2G**	2	477	423
**1.2G**	3	1787	1802
**3.1G**	1	1246	1224
**3.1G**	2	339	306
**3.1G**	3	887	641

Supplementary Table 3: Flight ability of SM9 transgenic pseudo-males versus WT males.

Line	Replicate	Number failed	Number successed
**WT**	1	55	31
**WT**	2	38	36
**WT**	3	79	11
**SM9**	1	35	41
**SM9**	2	34	65
**SM9**	3	50	14

Supplementary Table 4: Hatching rate of eggs from the SM9 versus WT lines

Replicate	Line	Larvae	Eggs
**1**	WT	373	641
**2**	WT	150	309
**3**	WT	115	201
**1**	SM9	138	286
**2**	SM9	82	147
**3**	SM9	153	264

Supplementary Table 5: Fertility of the SM9 versus WT lines

Replicate	Line	Number of progeny
**1**	WT	2224
**2**	WT	1400
**3**	WT	1581
**1**	SM9	2013
**2**	SM9	1479
**3**	SM9	858

Supplementary Table 6: Competitiveness of transgenic versus WT males

Comparison	Replicate	Number of transgenic progeny	Number of non-transgenic progeny
**SM9 vs WT**	1	22	364
**SM9 vs WT**	2	129	1912
**SM9 vs WT**	3	211	1540
**SM9 vs WT**	4	57	421
**SM9 vs WT**	5	157	1401
**1.2G vs WT**	1	586	2681
**1.2G vs WT**	2	530	4296
**1.2G vs WT**	3	474	3571
**1.2G vs WT**	4	187	1875
**3.1G vs WT**	1	16	281
**3.1G vs WT**	2	99	1042
**3.1G vs WT**	3	71	895
**3.1G vs WT**	4	175	1165
**3.1G vs WT**	5	73	710
