# Supplementary material for: Transgenic expression of Nix converts genetic females into males and allows automated sex sorting in Aedes albopictus
Source: Commun Biol. 2022 Mar 7;5:210. doi: 10.1038/s42003-022-03165-7 (PMC8901906; doi:10.1038/s42003-022-03165-7)
Supplement: Supplementary file 3 — Description of Additional Supplementary Files [file 42003_2022_3165_MOESM3_ESM.pdf]

Description of Additional Supplementary Files

File name: Supplementary Data 1
Description: Model output from all statistical analyses and performance assessment.

File name: Supplementary Data 2
Description: Wing length measurements (Fig. 3b).

File name: Supplementary Data 3
Description: qPCR data (Fig. 4).

File name: Supplementary Data 4
Description: COPAS data for SM9 line (Fig. 6a).

File name: Supplementary Data 5
Description: COPAS data for 1.2G line (Fig. 6b).

File name: Supplementary Data 6
Description: COPAS data for 3.1G line (Fig. 6c).
