# Supplementary material for: Transgenic expression of Nix converts genetic females into males and allows automated sex sorting in Aedes albopictus
Source: Commun Biol. 2022 Mar 7;5:210. doi: 10.1038/s42003-022-03165-7 (PMC8901906; doi:10.1038/s42003-022-03165-7)
Supplement: Supplementary file 4 — Supplementary Data 1 [file 42003_2022_3165_MOESM4_ESM.pdf]

Supplementary Information

Supplementary Data 1: Model output from all statistical analyses and performance assessment

Sex ratios

Goal

To evaluate whether the Nix-eGFP cassette was stable and adult males fully viable, we first determined the sex ratios of transgenic strains in comparison to that of the parental wild strain (WT).

Method

The effect of the lines on sex ratios was tested using linear generalised mixed-effect model and binomial distribution assumptions. The replicate was set as random effect as mesurements were performed on different days. The significance between the lines was tested by ANOVA followed by pairwise Tukey test.

Model output

```
##
##   Simultaneous Tests for General Linear Hypotheses
##
## Multiple Comparisons of Means: Tukey Contrasts
##
##
## Fit: glmer(formula = male_percentage ~ line + (1 | replicate), data = males,
##     family = binomial(link = logit))
##
## Linear Hypotheses:
##                   Estimate Std. Error z value Pr(>|z|)
## SM9 - BiA == 0     0.17455    0.08562   2.039    0.162
## 1.2G - BiA == 0  -0.11167    0.08380  -1.332    0.526
## 3.1G - BiA == 0   0.08180    0.08475   0.965    0.758
## 1.2G - SM9 == 0  -0.28622    0.04359  -6.566   <0.001 ***
## 3.1G - SM9 == 0  -0.09275    0.04498  -2.062    0.155
## 3.1G - 1.2G == 0  0.19347    0.03975   4.867   <0.001 ***
## ---
## Signif. codes:  0 '***' 0.001 '**' 0.01 '*' 0.05 '.' 0.1 ' ' 1
## (Adjusted p values reported -- single-step method)
```

```
##   Line  Estimate  Std_Error
## 1  BiA 0.5153738 0.08697104
## 2  SM9 0.5587437 0.04952702
## 3 1.2G 0.4874640 0.04510691
## 4 3.1G 0.5357665 0.04750739
```

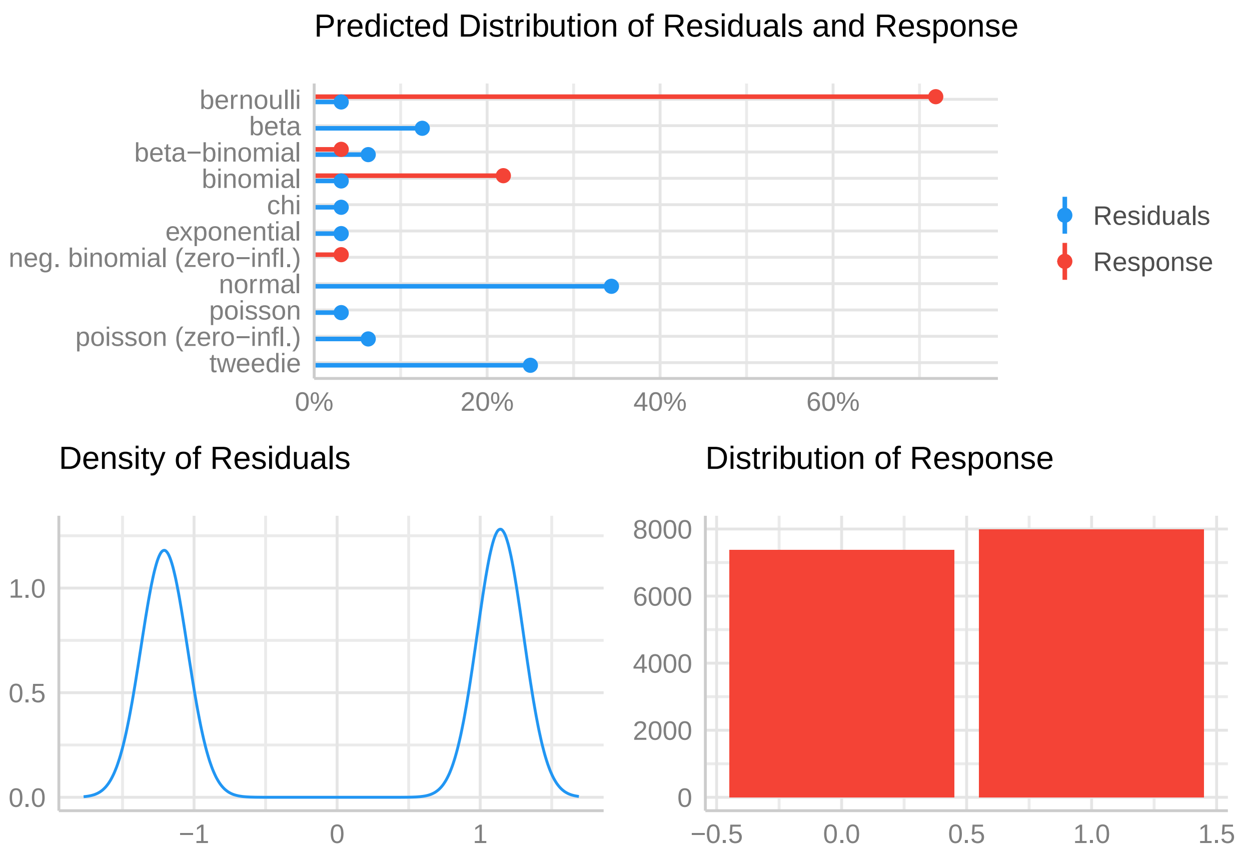

Predicted Distribution of Residuals and Response
bernoulli
beta
beta−binomial
binomial
chi
exponential
neg. binomial (zero−infl.)
normal
poisson
poisson (zero−infl.)
tweedie
0%
20%
40%
60%
Residuals
Response
Density of Residuals
1.0
0.5
0.0
−1
0
1
Distribution of Response
8000
6000
4000
2000
0
−0.5
0.0
0.5
1.0
1.5

Wing lengths

Goal

In *Aedes* mosquitoes, males and females display a significant size dimorphism, with females having a larger body size. We compared the body size of masculinized genetic females to WT females and WT males using wing length as a proxy for mosquito body size.

Method

The effect of lines on wing length was tested using linear model and normal distribution assumptions. The significance between the lines was tested by ANOVA followed by pairwise Tukey test.

Model output

```
##
## Call:
## lm(formula = Length ~ Line, data = wings)
##
## Residuals:
##     Min      1Q  Median      3Q     Max
## -71.257 -15.393  -1.055  16.187 127.278
##
## Coefficients:
##              Estimate Std. Error t value Pr(>|t|)
## (Intercept)   815.065      4.469  182.40   <2e-16 ***
## LineBiA male -109.314      6.361  -17.18   <2e-16 ***
## LineSM9 male -108.970      6.016  -18.11   <2e-16 ***
## ---
## Signif. codes:  0 '***' 0.001 '**' 0.01 '*' 0.05 '.' 0.1 ' ' 1
##
## Residual standard error: 27.91 on 122 degrees of freedom
## Multiple R-squared:  0.7708, Adjusted R-squared:  0.767
## F-statistic: 205.1 on 2 and 122 DF,  p-value: < 2.2e-16
```

```
##   Tukey multiple comparisons of means
##     95% family-wise confidence level
##
## Fit: aov(formula = mod.wings)
##
## $Line
##                              diff        lwr       upr     p adj
## BiA male-BiA female -109.3141235 -124.40642 -94.22182 0.0000000
## SM9 male-BiA female -108.9702244 -123.24407 -94.69638 0.0000000
## SM9 male-BiA male      0.3438991  -14.03319  14.72099 0.9982258
```

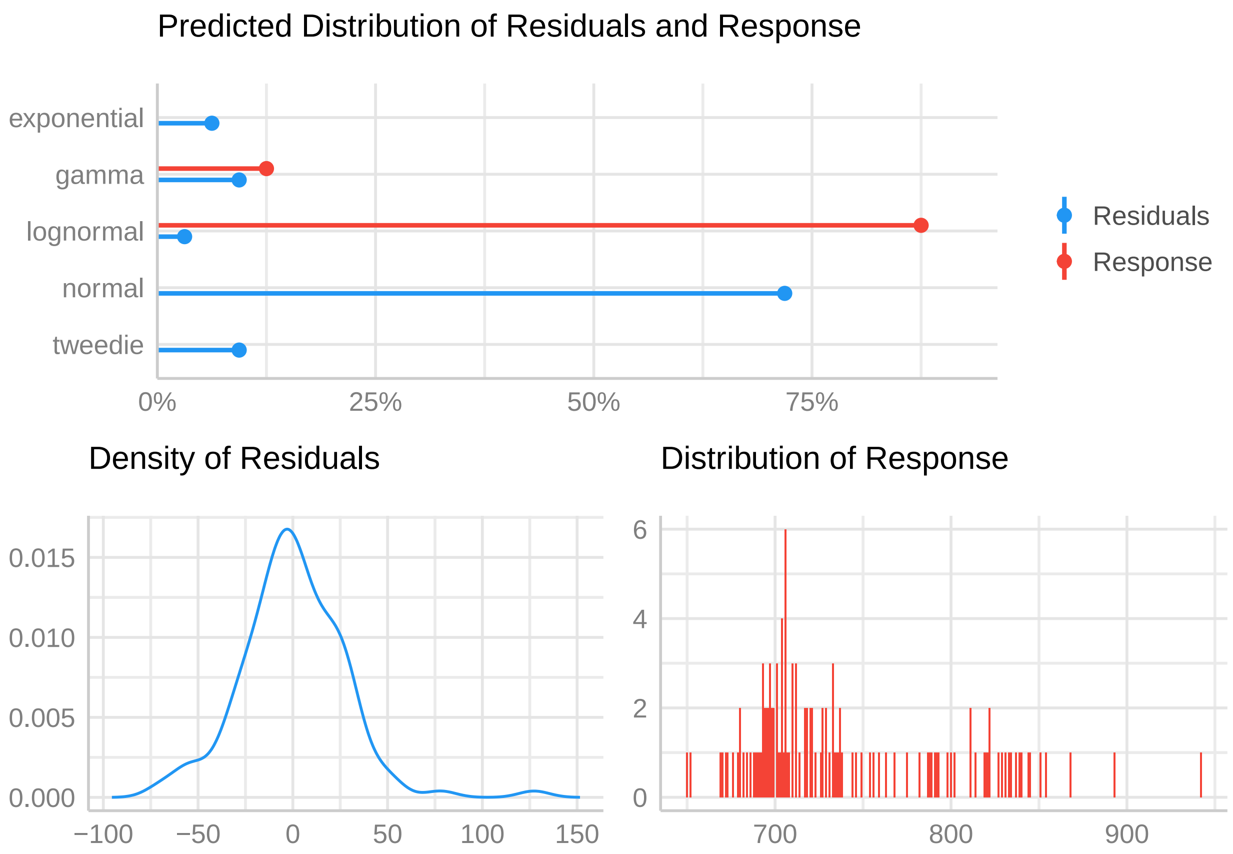
Predicted Distribution of Residuals and Response
exponential
gamma
lognormal
normal
tweedie
0%
25%
50%
75%
Residuals
Response
Density of Residuals
0.015
0.010
0.005
0.000
−100
−50
0
50
100
150
Distribution of Response
6
4
2
0
700
800
900

RT-qPCR : relative expresion of *Nix*

Goal

To assess whether transgenic males express *Nix* in similar levels to wild-type males, expression levels were measured by RT-qPCR at the pupal stage.

Method

For qPCR, three technical replicates of each biological sample were used. Each line was tested on 3 independent biological samples. To test the effects of the lines on gene expression we used linear model and normal distribution assumptions.The significance between the lines was tested by ANOVA followed by pairwise Tukey test.

Model output

```
##   Tukey multiple comparisons of means
##     95% family-wise confidence level
##
## Fit: aov(formula = mod.dCt)
##
## $line
##                             diff         lwr       upr     p adj
## WT female-WT male   -10.65328068 -15.9581933 -5.348368 0.0005953
## SM9 male-WT male     -2.92265574  -8.8537284  3.008417 0.5014045
## 1.2G male-WT male    -2.96413962  -8.2690522  2.340773 0.3910419
## 3.1G male-WT male     2.25456640  -3.0503462  7.559479 0.6262636
## SM9 male-WT female    7.73062494   1.7995523 13.661698 0.0115720
## 1.2G male-WT female   7.68914106   2.3842284 12.994054 0.0059238
## 3.1G male-WT female  12.90784709   7.6029345 18.212760 0.0001348
## 1.2G male-SM9 male   -0.04148388  -5.9725565  5.889589 0.9999999
## 3.1G male-SM9 male    5.17722214  -0.7538505 11.108295 0.0933632
## 3.1G male-1.2G male   5.21870602  -0.0862066 10.523619 0.0541624
```

```
## [1] 3406.05
```

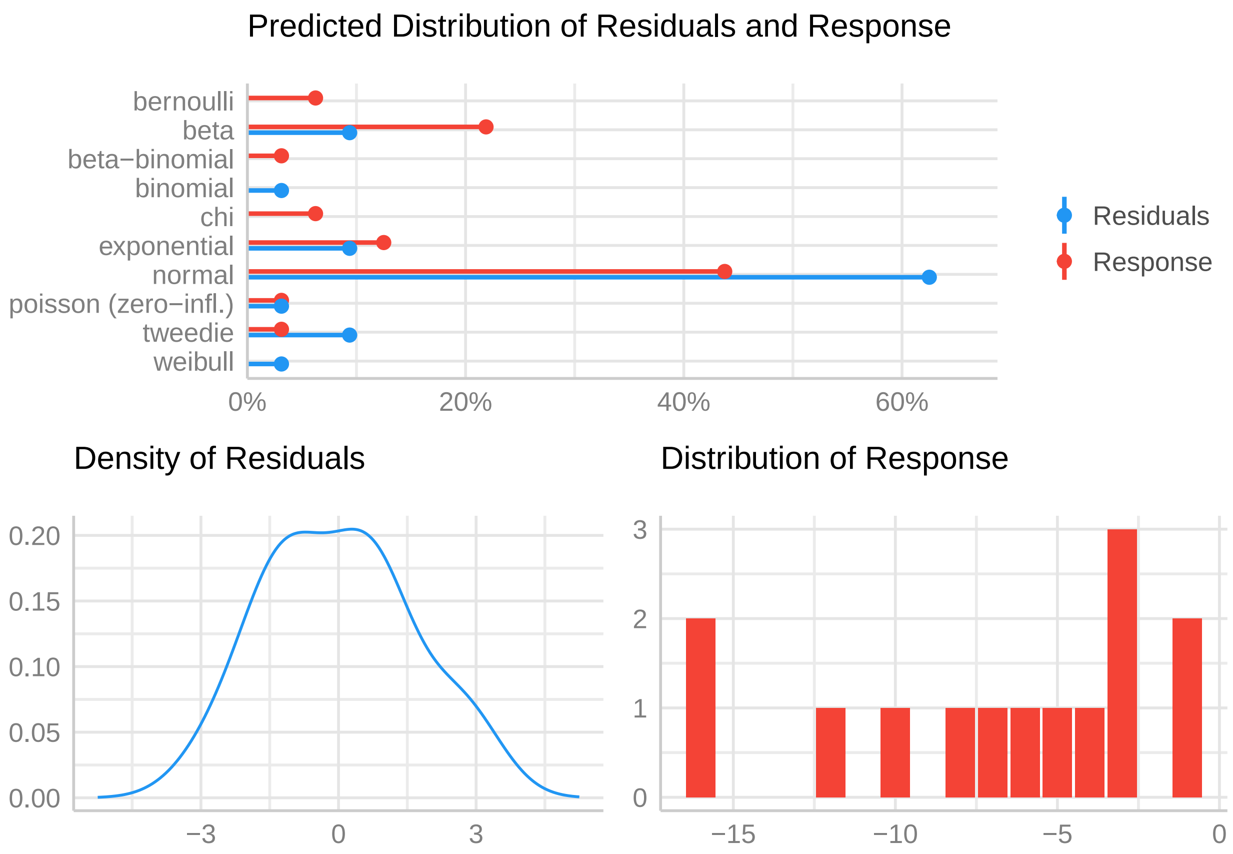
Predicted Distribution of Residuals and Response
bernoulli
beta
beta−binomial
binomial
chi
exponential
normal
poisson (zero−infl.)
tweedie
weibull
0%
20%
40%
60%
Residuals
Response
Density of Residuals
0.20
0.15
0.10
0.05
0.00
−3
0
3
Distribution of Response
3
2
1
0
−15
−10
−5
0

RT-qPCR : relative expresion of *myo-sex*

Goal

To assess whether transgenic males express *myo-sex* in similar levels to wild-type males, expression levels were measured by RT-qPCR at the pupal stage.

Method

For qPCR, three technical replicates of each biological sample were used. Each line was tested on 3 independent biological samples. To test the effects of the lines on gene expression we used linear model and normal distribution assumptions.The significance between the lines was tested by ANOVA followed by pairwise Tukey test.

Model output

```
##   Tukey multiple comparisons of means
##     95% family-wise confidence level
##
## Fit: aov(formula = mod.dCt)
##
## $line
##                             diff       lwr       upr     p adj
## WT female-WT male    -4.50142023 -6.865756 -2.137084 0.0008858
## SM9 male-WT male      0.07875527 -2.564652  2.722163 0.9999723
## 1.2G male-WT male     0.46634928 -1.897986  2.830685 0.9596094
## 3.1G male-WT male     0.71218332 -1.652152  3.076519 0.8434409
## SM9 male-WT female    4.58017550  1.936768  7.223583 0.0017537
## 1.2G male-WT female   4.96776952  2.603434  7.332105 0.0004226
## 3.1G male-WT female   5.21360355  2.849268  7.577939 0.0002916
## 1.2G male-SM9 male    0.38759401 -2.255814  3.031002 0.9860491
## 3.1G male-SM9 male    0.63342805 -2.009980  3.276836 0.9223992
## 3.1G male-1.2G male   0.24583403 -2.118502  2.610170 0.9961853

## [1] 32.59559
```

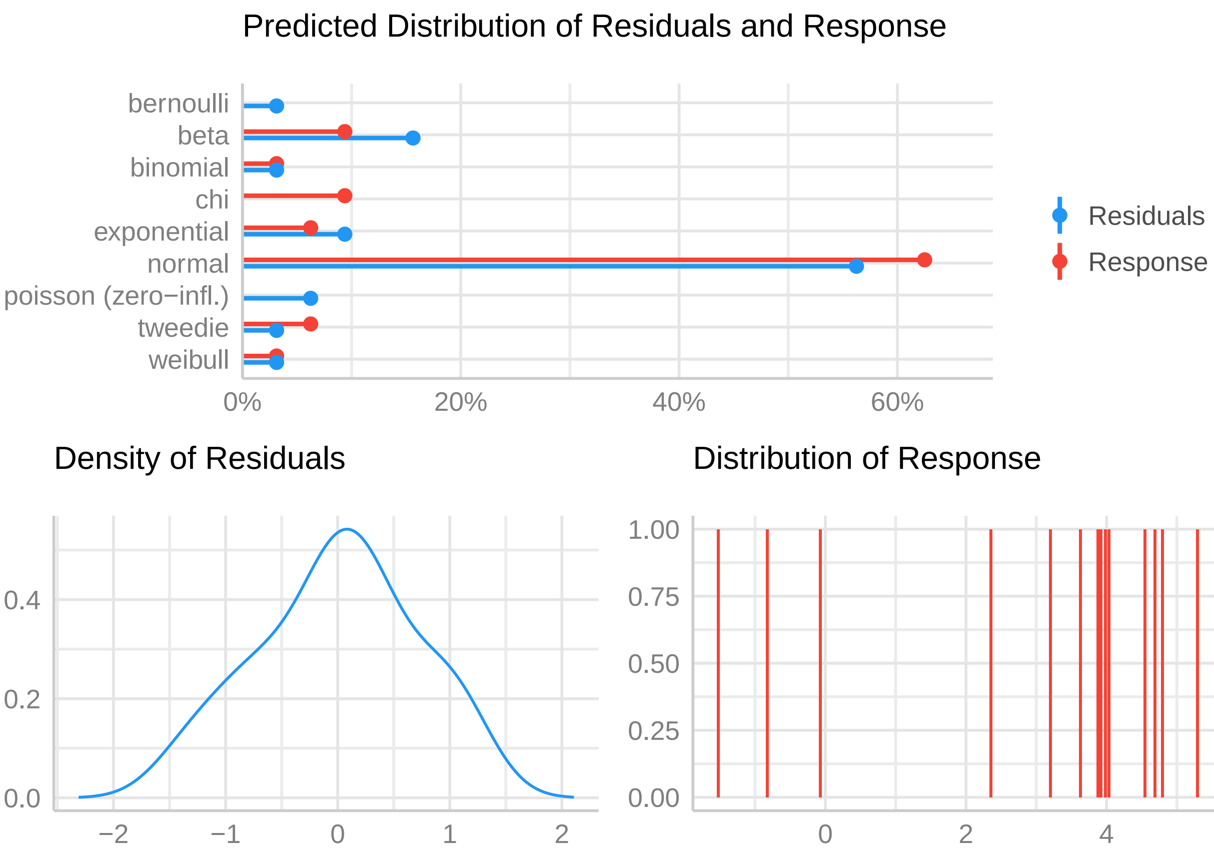
Predicted Distribution of Residuals and Response
bernoulli
beta
binomial
chi
exponential
normal
poisson (zero−infl.)
tweedie
weibull
0%
20%
40%
60%
Residuals
Response
Density of Residuals
0.4
0.2
0.0
−2
−1
0
1
2
Distribution of Response
1.00
0.75
0.50
0.25
0.00
0
2
4

RT-qPCR : relative expresion of LOC109402113

Goal

To assess whether transgenic males express LOC109402113 (*Ae. aegypti myo-fem* homologue) in similar levels to wild-type males, expression levels were measured by RT-qPCR at the pupal stage.

Method

For qPCR, three technical replicates of each biological sample were used. Each line was tested on 3 independent biological samples. To test the effects of the lines on gene expression we used linear model and normal distribution assumptions.The significance between the lines was tested by ANOVA followed by pairwise Tukey test.

Model output

```
##   Tukey multiple comparisons of means
##     95% family-wise confidence level
##
## Fit: aov(formula = mod.dCt)
##
## $line
##                           diff        lwr       upr     p adj
## WT female-WT male    12.4843140   9.035306 15.933322 0.0000051
## SM9 male-WT male      0.4245338  -3.431574  4.280642 0.9952514
## 1.2G male-WT male     1.0438734  -2.405134  4.492881 0.8412790
## 3.1G male-WT male     1.4883477  -1.960660  4.937356 0.6139971
## SM9 male-WT female  -12.0597801 -15.915888 -8.203672 0.0000174
## 1.2G male-WT female -11.4404406 -14.889448 -7.991433 0.0000106
## 3.1G male-WT female -10.9959663 -14.444974 -7.546958 0.0000148
## 1.2G male-SM9 male    0.6193395  -3.236768  4.475448 0.9805259
## 3.1G male-SM9 male    1.0638138  -2.792294  4.919922 0.8792667
## 3.1G male-1.2G male   0.4444743  -3.004534  3.893482 0.9913742

## [1] 3947.643
```

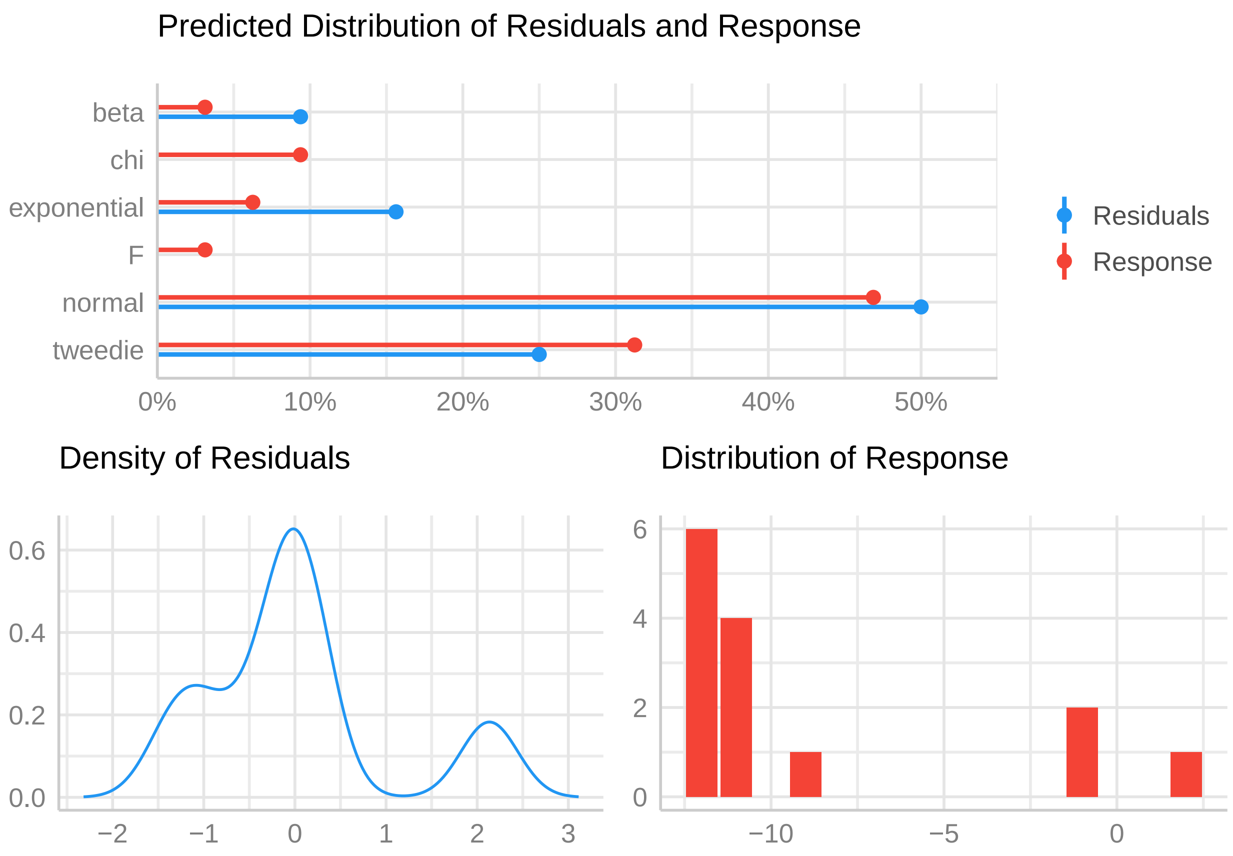
Predicted Distribution of Residuals and Response
beta
chi
exponential
F
normal
tweedie
0%
10%
20%
30%
40%
50%
Residuals
Response
Density of Residuals
0.6
0.4
0.2
0.0
−2
−1
0
1
2
3
Distribution of Response
6
4
2
0
−10
−5
0

RT-qPCR : relative expresion of LOC115254984

Goal

To assess whether transgenic males express LOC115254984 (*Ae. aegypti myo-fem homologue*) in similar levels to wild-type males, expression levels were measured by RT-qPCR at the pupal stage.

Method

For qPCR, three technical replicates of each biological sample were used. Each line was tested on 3 independent biological samples. To test the effects of the lines on gene expression we used linear model and normal distribution assumptions.The significance between the lines was tested by ANOVA followed by pairwise Tukey test.

Model output

```
##   Tukey multiple comparisons of means
##     95% family-wise confidence level
##
## Fit: aov(formula = mod.dCt)
##
## $line
##                            diff        lwr       upr     p adj
## WT female-WT male    12.6736647   7.185298 18.162031 0.0003073
## SM9 male-WT male     -1.1926133  -7.328794  4.943567 0.9573246
## 1.2G male-WT male     2.1106772  -4.025503  8.246858 0.7580825
## 3.1G male-WT male     0.6872514  -4.801115  6.175618 0.9912738
## SM9 male-WT female  -13.8662780 -20.002459 -7.730097 0.0003583
## 1.2G male-WT female -10.5629875 -16.699168 -4.426807 0.0022847
## 3.1G male-WT female -11.9864133 -17.474780 -6.498047 0.0004555
## 1.2G male-SM9 male    3.3032905  -3.418558 10.025140 0.4846920
## 3.1G male-SM9 male    1.8798647  -4.256316  8.016045 0.8221682
## 3.1G male-1.2G male  -1.4234258  -7.559606  4.712755 0.9230466
```

```
## [1] 7440.647
```

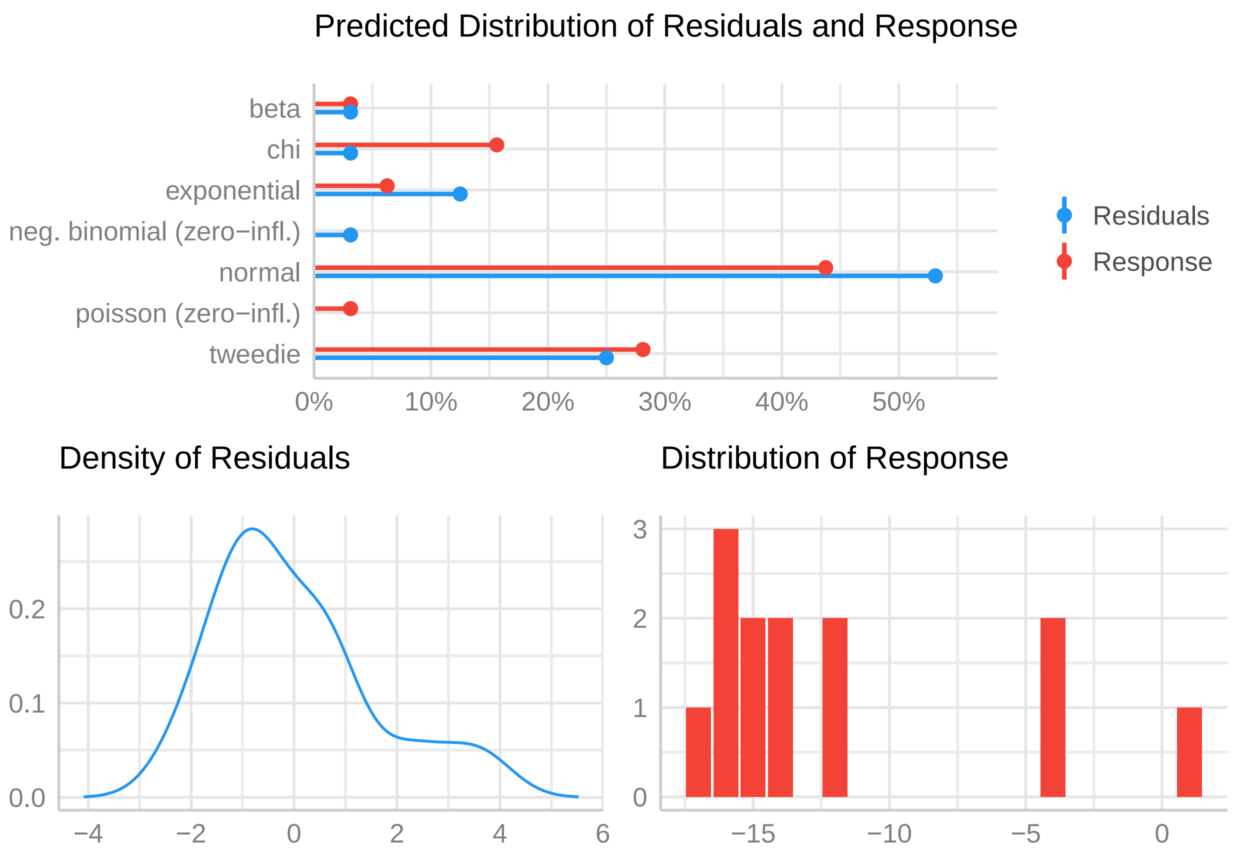
Predicted Distribution of Residuals and Response
beta
chi
exponential
neg. binomial (zero−infl.)
normal
poisson (zero−infl.)
tweedie
0%
10%
20%
30%
40%
50%
Residuals
Response
Density of Residuals
0.2
0.1
0.0
−4
−2
0
2
4
6
Distribution of Response
3
2
1
0
−15
−10
−5
0

Flight ability

Goal

Since we observed that genes potentially involved in flight were regulated similarly in *Nix*-expressing pseudo-males comparing to wild-type counterparts, we compared SM9 males' flight ability to WT males by performing a flight test.

Method

The effect of the lines on flight ability was tested using linear generalized mixed-effect model and binomial distribution assumptions. The replicate was set as random effect for flight tests as experiments was performed on different days.

Model output

```
## Generalized linear mixed model fit by maximum likelihood (Laplace
##   Approximation) [glmerMod]
##  Family: binomial  ( logit )
## Formula: escape.rate ~ line + (1 | replicate)
##    Data: flight
##
##      AIC      BIC   logLik deviance df.resid
##    599.5    612.0   -296.7    593.5      486
##
## Scaled residuals:
##     Min      1Q  Median      3Q     Max
## -1.3666 -0.7490 -0.3862  0.9328  2.5890
##
## Random effects:
##  Groups    Name        Variance Std.Dev.
##  replicate (Intercept) 0.625    0.7906
## Number of obs: 489, groups:  replicate, 3
##
## Fixed effects:
##             Estimate Std. Error z value Pr(>|z|)
## (Intercept)  -0.8617     0.4796  -1.797 0.072351 .
## lineSM9       0.7171     0.1996   3.593 0.000327 ***
## ---
## Signif. codes:  0 '***' 0.001 '**' 0.01 '*' 0.05 '.' 0.1 ' ' 1
##
## Correlation of Fixed Effects:
##         (Intr)
## lineSM9 -0.217
```

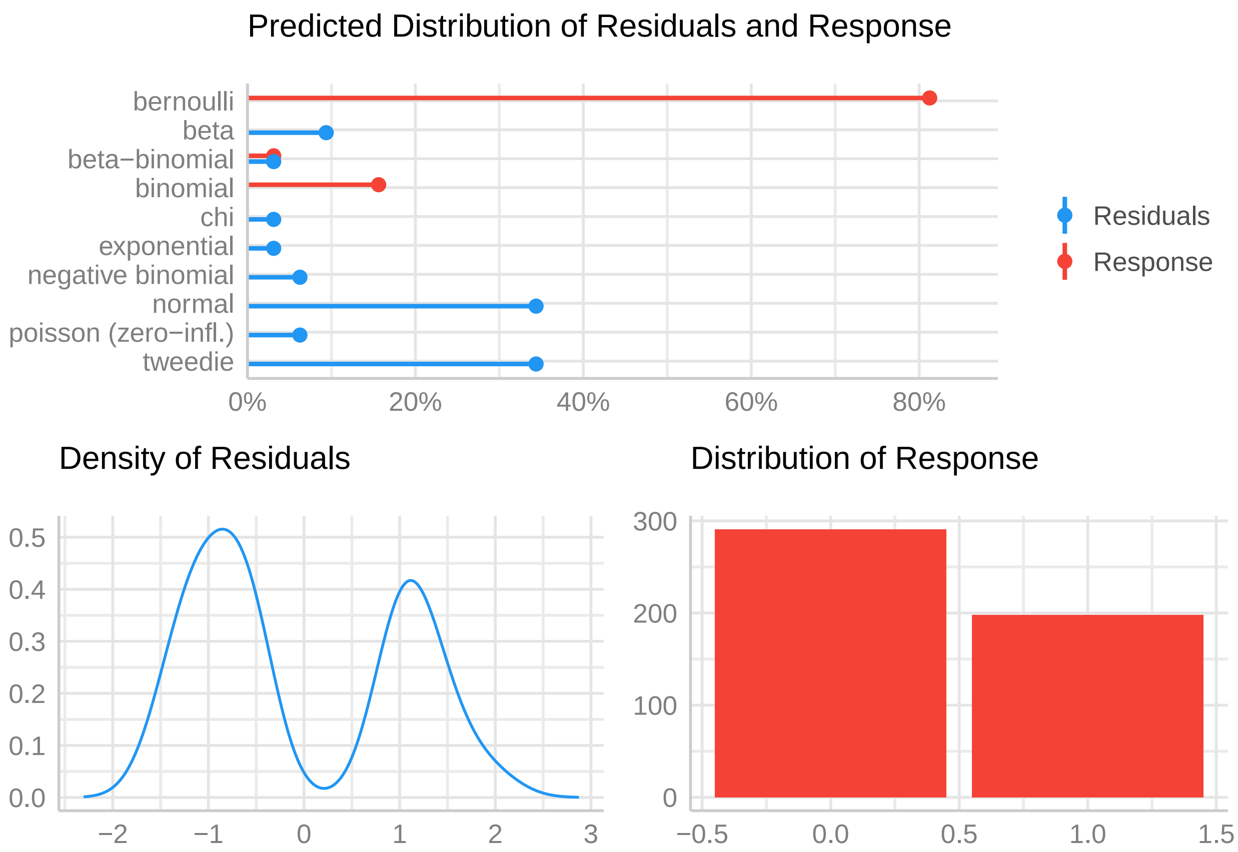
Predicted Distribution of Residuals and Response
bernoulli
beta
beta−binomial
binomial
chi
exponential
negative binomial
normal
poisson (zero−infl.)
tweedie
0%
20%
40%
60%
80%
Residuals
Response
Density of Residuals
0.5
0.4
0.3
0.2
0.1
0.0
−2
−1
0
1
2
3
Distribution of Response
300
200
100
0
−0.5
0.0
0.5
1.0
1.5

Hatching rate

Goal

We compared the hatching rate of eggs produced by the trasngenic SM9 line and the control wild-type line.

Method

The effect of the lines on hatching rates was tested using linear generalized mixed-effect model and binomial distribution assumptions.

Model output

```
##
## Call:
## glm(formula = "hatched ~ line", family = binomial(link = logit),
##     data = hatch)
##
## Deviance Residuals:
##    Min      1Q  Median      3Q     Max
## -1.271  -1.271   1.086   1.086   1.118
##
## Coefficients:
##             Estimate Std. Error z value Pr(>|z|)
## (Intercept)  0.21806    0.05930   3.677 0.000236 ***
## lineSM9     -0.07723    0.09635  -0.802 0.422842
## ---
## Signif. codes:  0 '***' 0.001 '**' 0.01 '*' 0.05 '.' 0.1 ' ' 1
##
## (Dispersion parameter for binomial family taken to be 1)
##
##     Null deviance: 2545.5  on 1847  degrees of freedom
## Residual deviance: 2544.8  on 1846  degrees of freedom
## AIC: 2548.8
##
## Number of Fisher Scoring iterations: 3
```

```
## # A tibble: 2 x 3
##   line  estimate     SE
##   <chr>    <dbl>  <dbl>
## 1 BiA      0.554 0.0147
## 2 SM9      0.535 0.0189
```

```
## Warning: Removed 9 rows containing missing values (geom_segment).
```

```
## Warning: Removed 9 rows containing missing values (geom_point).
```

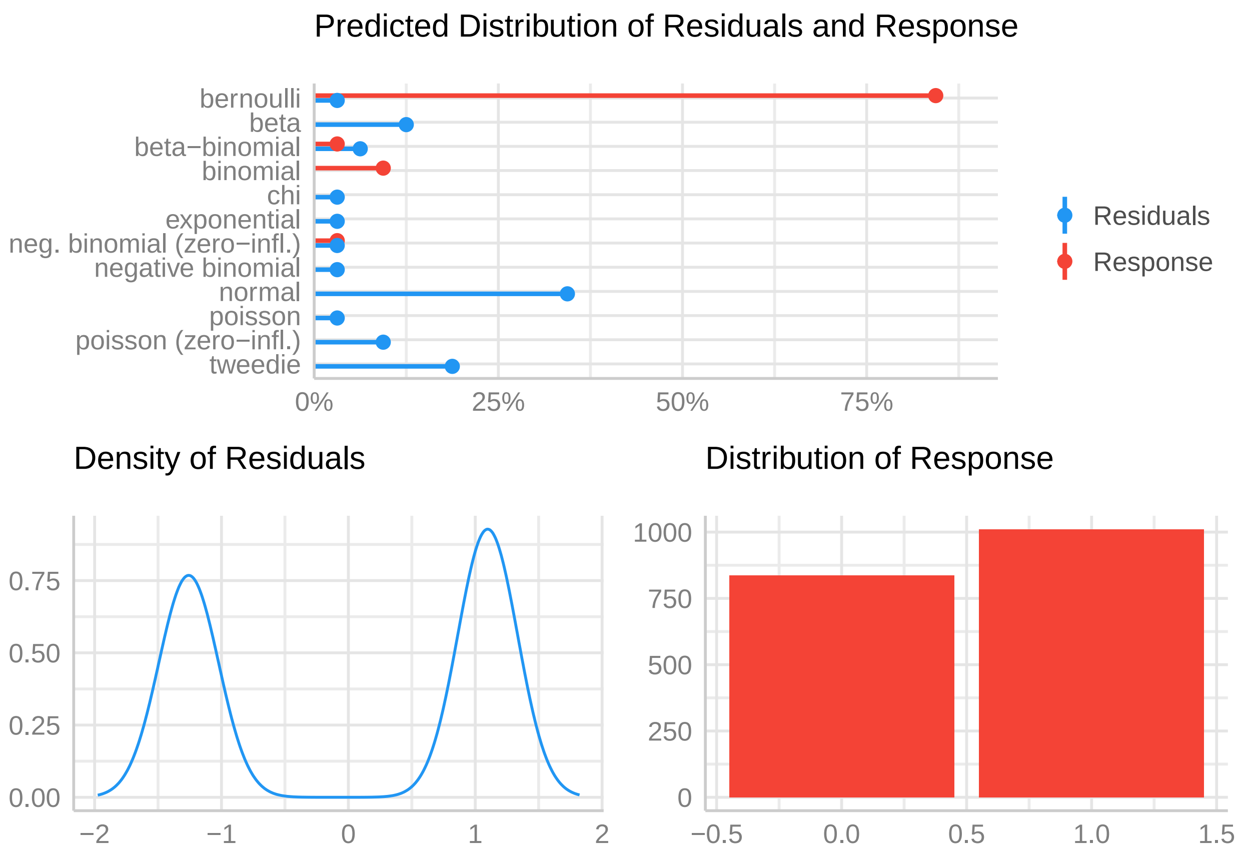
Predicted Distribution of Residuals and Response
bernoulli
beta
beta−binomial
binomial
chi
exponential
neg. binomial (zero−infl.)
negative binomial
normal
poisson
poisson (zero−infl.)
tweedie
0%
25%
50%
75%
Residuals
Response
Density of Residuals
0.75
0.50
0.25
0.00
−2
−1
0
1
2
Distribution of Response
1000
750
500
250
0
−0.5
0.0
0.5
1.0
1.5

Fertility

Goal

We compared the fertility (total number of progeny obtained from a given number of females) of the SM9 line and the wild-type line.

Method

The effect of lines on fertility was tested using linear model and normal distribution assumptions.

Model output

```
##
## Call:
## lm(formula = "fertility ~ Line", data = fertil)
##
## Residuals:
##    1    2    3    4    5    6
##  489 -335 -154  563   29 -592
##
## Coefficients:
##             Estimate Std. Error t value Pr(>|t|)
## (Intercept)   1450.0      294.9   4.918  0.00794 **
## LineWT         285.0      417.0   0.683  0.53186
## ---
## Signif. codes:  0 '***' 0.001 '**' 0.01 '*' 0.05 '.' 0.1 ' ' 1
##
## Residual standard error: 510.7 on 4 degrees of freedom
## Multiple R-squared:  0.1046, Adjusted R-squared:  -0.1193
## F-statistic: 0.4671 on 1 and 4 DF,  p-value: 0.5319
```

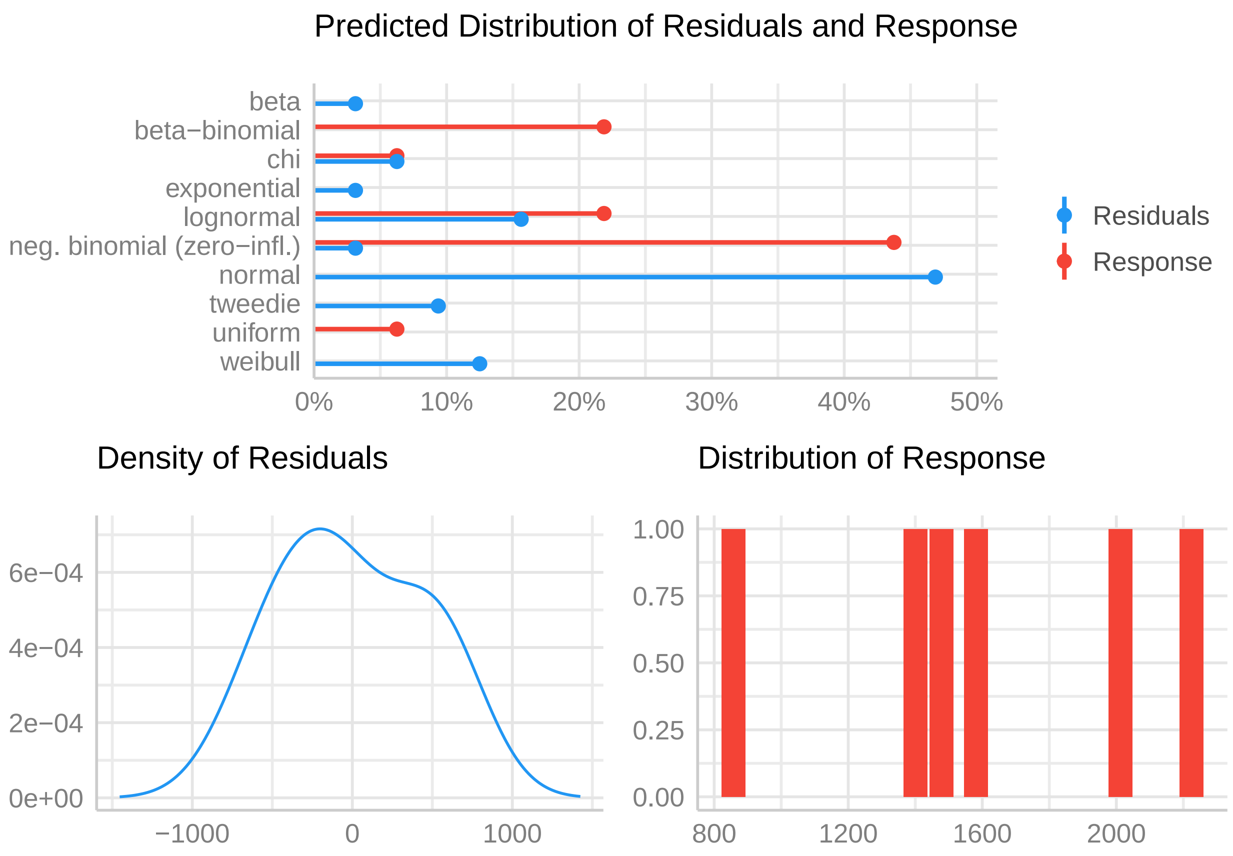

Predicted Distribution of Residuals and Response
beta
beta−binomial
chi
exponential
lognormal
neg. binomial (zero−infl.)
normal
tweedie
uniform
weibull
0%
10%
20%
30%
40%
50%
Residuals
Response
Density of Residuals
6e−04
4e−04
2e−04
0e+00
−1000
0
1000
Distribution of Response
1.00
0.75
0.50
0.25
0.00
800
1200
1600
2000

Relative competitiveness of SM9 compared to WT

Goal

We measured relative competitiveness between SM9 males and wild-type males by mixing equal numbers of transgenic and wild-type males with wild-type females. The percentage of transgenic progeny was used as an indicator for relative competitiveness.

Method

The effect of the lines on competitiveness was tested using linear generalised model and binomial distribution assumptions. For this test, we compared the number of SM9 male progeny measured in the offspring of each competitiveness replicate, to the expected number of progeny that would have been obtained if both lines were as competitive.

Model output

```
##
## Call:
## glm(formula = "result ~ line", family = binomial(link = logit),
##     data = compet.Bernou)
##
## Deviance Residuals:
##     Min       1Q   Median       3Q      Max
## -0.8086  -0.8086  -0.4644  -0.4644   2.1357
##
## Coefficients:
##             Estimate Std. Error z value Pr(>|z|)
## (Intercept) -0.95011    0.02972  -31.97   <2e-16 ***
## lineobs     -1.22271    0.05307  -23.04   <2e-16 ***
## ---
## Signif. codes:  0 '***' 0.001 '**' 0.01 '*' 0.05 '.' 0.1 ' ' 1
##
## (Dispersion parameter for binomial family taken to be 1)
##
##     Null deviance: 10971  on 11264  degrees of freedom
## Residual deviance: 10383  on 11263  degrees of freedom
## AIC: 10387
##
## Number of Fisher Scoring iterations: 4
```

```
## # A tibble: 2 x 3
##   line  estimate      SE
##   <fct>    <dbl>   <dbl>
## 1 th       0.279 0.00598
## 2 obs      0.102 0.00404
```

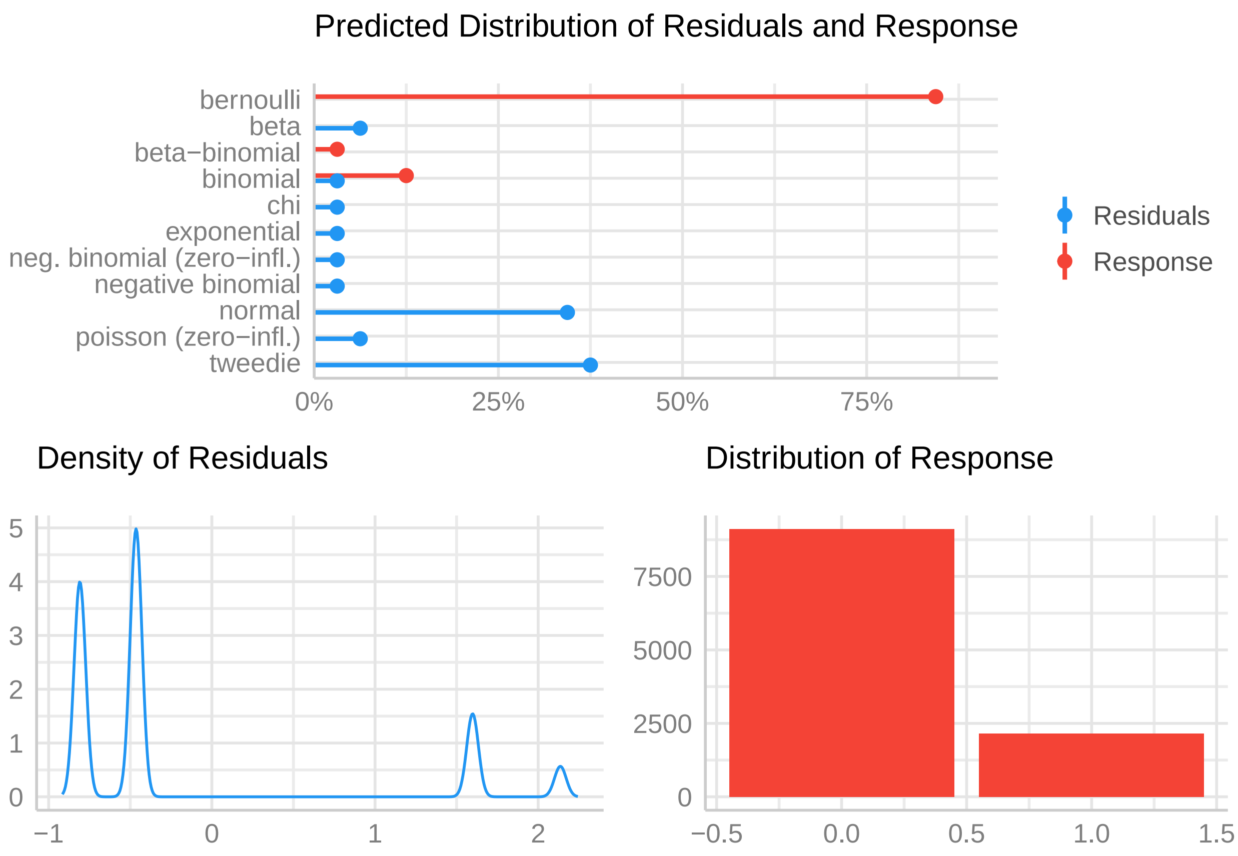
Predicted Distribution of Residuals and Response
bernoulli
beta
beta−binomial
binomial
chi
exponential
neg. binomial (zero−infl.)
negative binomial
normal
poisson (zero−infl.)
tweedie
0%
25%
50%
75%
Residuals
Response
Density of Residuals
5
4
3
2
1
0
−1
0
1
2
Distribution of Response
7500
5000
2500
0
−0.5
0.0
0.5
1.0
1.5

Relative competitiveness of 1.2G compared to WT

Goal

We measured relative competitiveness between 1.2G males and wild-type males by mixing equal numbers of transgenic and wild-type males with wild-type females. The percentage of transgenic progeny was used as an indicator for relative competitiveness.

Method

The effect of the lines on competitiveness was tested using linear generalised model and binomial distribution assumptions. For this test, we compared the number of 1.2G male progeny measured in the offspring of each competitiveness replicate, to the expected number of progeny that would have been obtained if both lines were as competitive.

Model output

```
##
## Call:
## glm(formula = "result ~ line", family = binomial(link = logit),
##     data = compet1.2g.Bernou)
##
## Deviance Residuals:
##     Min       1Q   Median       3Q      Max
## -0.7497  -0.7497  -0.5171  -0.5171   2.0388
##
## Coefficients:
##             Estimate Std. Error z value Pr(>|z|)
## (Intercept) -1.12547    0.01951  -57.67   <2e-16 ***
## lineobs     -0.81916    0.03200  -25.60   <2e-16 ***
## ---
## Signif. codes:  0 '***' 0.001 '**' 0.01 '*' 0.05 '.' 0.1 ' ' 1
##
## (Dispersion parameter for binomial family taken to be 1)
##
##     Null deviance: 27202  on 28395  degrees of freedom
## Residual deviance: 26516  on 28394  degrees of freedom
## AIC: 26520
##
## Number of Fisher Scoring iterations: 4
```

```
## # A tibble: 2 x 3
##   line  estimate      SE
##   <fct>    <dbl>   <dbl>
## 1 th       0.245 0.00361
## 2 obs      0.125 0.00278
```

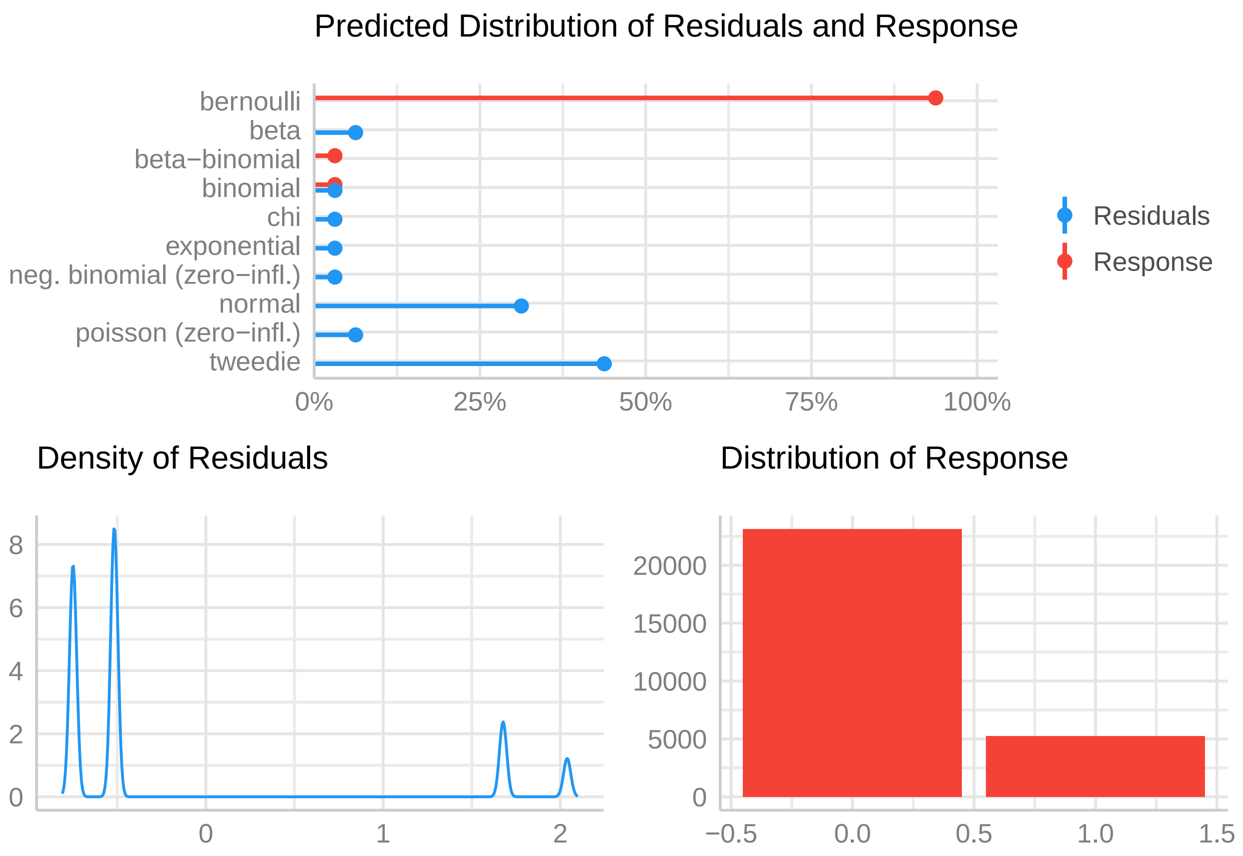
Predicted Distribution of Residuals and Response
bernoulli
beta
beta−binomial
binomial
chi
exponential
neg. binomial (zero−infl.)
normal
poisson (zero−infl.)
tweedie
0%
25%
50%
75%
100%
Residuals
Response
Density of Residuals
8
6
4
2
0
0
1
2
Distribution of Response
20000
15000
10000
5000
0
−0.5
0.0
0.5
1.0
1.5

Relative competitiveness of 3.1G compared to WT

Goal

We measured relative competitiveness between 3.1G males and wild-type males by mixing equal numbers of transgenic and wild-type males with wild-type females. The percentage of transgenic progeny was used as an indicator for relative competitiveness.

Method

The effect of the lines on competitiveness was tested using linear generalised model and binomial distribution assumptions. For this test, we compared the number of 3.1G male progeny measured in the offspring of each competitiveness replicate, to the expected number of progeny that would have been obtained if both lines were as competitive.

Model output

```
##
## Call:
## glm(formula = "result ~ line", family = binomial(link = logit),
##     data = compet3.1g.Bernou)
##
## Deviance Residuals:
##    Min      1Q  Median      3Q     Max
## -0.793  -0.793  -0.449  -0.449   2.166
##
## Coefficients:
##             Estimate Std. Error z value Pr(>|z|)
## (Intercept) -0.99568    0.03350  -29.72   <2e-16 ***
## lineobs     -1.24831    0.06058  -20.61   <2e-16 ***
## ---
## Signif. codes:  0 '***' 0.001 '**' 0.01 '*' 0.05 '.' 0.1 ' ' 1
##
## (Dispersion parameter for binomial family taken to be 1)
##
##     Null deviance: 8607.2  on 9048  degrees of freedom
## Residual deviance: 8133.4  on 9047  degrees of freedom
## AIC: 8137.4
##
## Number of Fisher Scoring iterations: 4
```

```
## # A tibble: 2 x 3
##   line  estimate      SE
##   <fct>    <dbl>   <dbl>
## 1 th      0.270  0.00660
## 2 obs     0.0959 0.00438
```

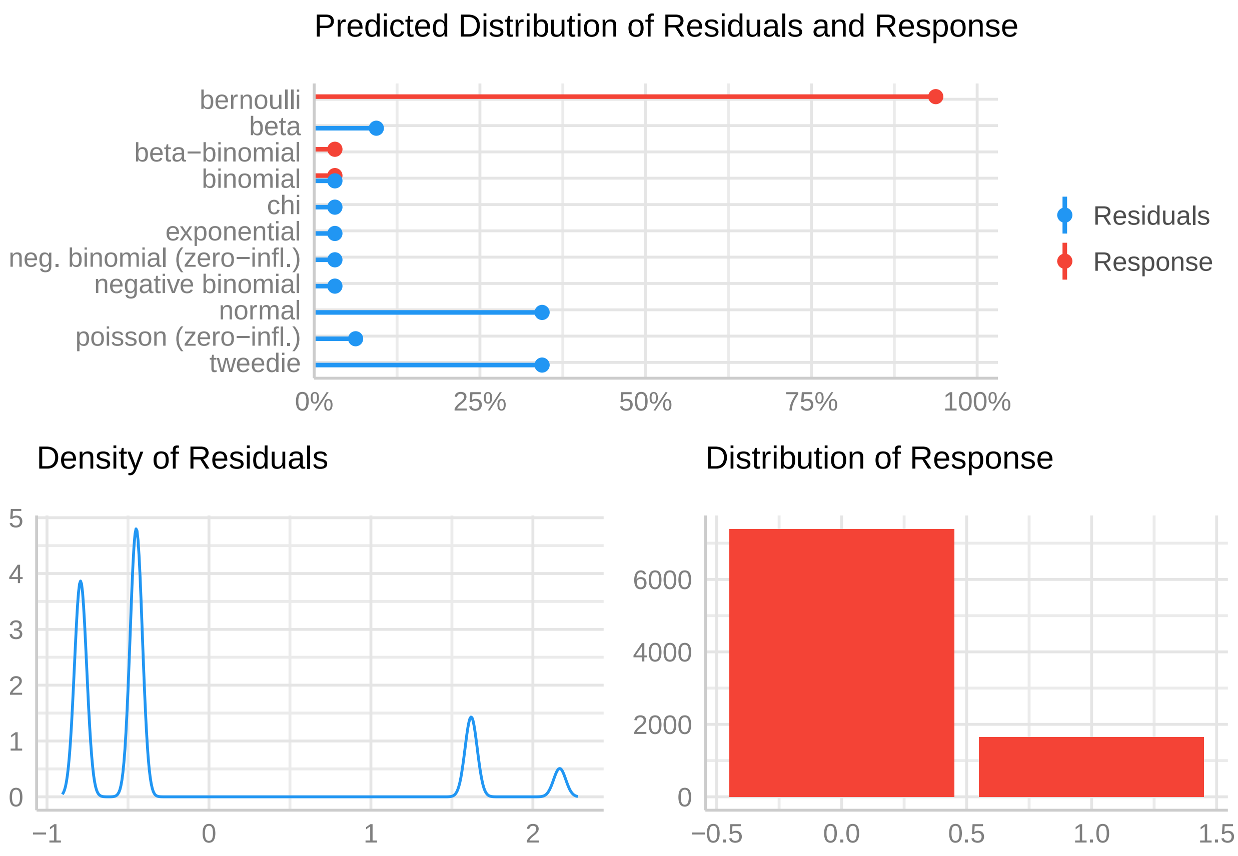
Predicted Distribution of Residuals and Response
bernoulli
beta
beta−binomial
binomial
chi
exponential
neg. binomial (zero−infl.)
negative binomial
normal
poisson (zero−infl.)
tweedie
0%
25%
50%
75%
100%
Residuals
Response
Density of Residuals
5
4
3
2
1
0
−1
0
1
2
Distribution of Response
6000
4000
2000
0
−0.5
0.0
0.5
1.0
1.5
