# Supplementary material for: Transgenic expression of Nix converts genetic females into males and allows automated sex sorting in Aedes albopictus
Source: Commun Biol. 2022 Mar 7;5:210. doi: 10.1038/s42003-022-03165-7 (PMC8901906; doi:10.1038/s42003-022-03165-7)
Supplement: Supplementary file 10 — Reporting Summary [file 42003_2022_3165_MOESM10_ESM.pdf]

nature portfolio

Corresponding author(s): Célia Lutrat, celia.lutrat@outlook.com
Eric Marois, e.marois@unistra.fr

Last updated by author(s): Nov. 16, 2021

Reporting Summary

Nature Portfolio wishes to improve the reproducibility of the work that we publish. This form provides structure for consistency and transparency in reporting. For further information on Nature Portfolio policies, see our Editorial Policies and the Editorial Policy Checklist.

Statistics

For all statistical analyses, confirm that the following items are present in the figure legend, table legend, main text, or Methods section.

n/a	Confirmed	
☐	☒	The exact sample size (*n*) for each experimental group/condition, given as a discrete number and unit of measurement
☐	☒	A statement on whether measurements were taken from distinct samples or whether the same sample was measured repeatedly
☐	☒	The statistical test(s) used AND whether they are one- or two-sided<br>*Only common tests should be described solely by name; describe more complex techniques in the Methods section.*
☒	☐	A description of all covariates tested
☐	☒	A description of any assumptions or corrections, such as tests of normality and adjustment for multiple comparisons
☐	☒	A full description of the statistical parameters including central tendency (e.g. means) or other basic estimates (e.g. regression coefficient) AND variation (e.g. standard deviation) or associated estimates of uncertainty (e.g. confidence intervals)
☐	☒	For null hypothesis testing, the test statistic (e.g. *F*, *t*, *r*) with confidence intervals, effect sizes, degrees of freedom and *P* value noted<br>*Give P values as exact values whenever suitable.*
☒	☐	For Bayesian analysis, information on the choice of priors and Markov chain Monte Carlo settings
☒	☐	For hierarchical and complex designs, identification of the appropriate level for tests and full reporting of outcomes
☒	☐	Estimates of effect sizes (e.g. Cohen's *d*, Pearson's *r*), indicating how they were calculated

Our web collection on statistics for biologists contains articles on many of the points above.

Software and code

Policy information about availability of computer code

Data collection	COPAS measurements were performed on Union Biometrica BIOSORT software. qPCR data were collected on the Applied Biosystems 7500 software v2.3.
Data analysis	Data analysis was performed on R version 4.0.5.

For manuscripts utilizing custom algorithms or software that are central to the research but not yet described in published literature, software must be made available to editors and reviewers. We strongly encourage code deposition in a community repository (e.g. GitHub). See the Nature Portfolio guidelines for submitting code & software for further information.

Data

Policy information about availability of data

All manuscripts must include a data availability statement. This statement should provide the following information, where applicable:

- Accession codes, unique identifiers, or web links for publicly available datasets
- A description of any restrictions on data availability
- For clinical datasets or third party data, please ensure that the statement adheres to our policy

Plasmid sequences have been deposited on Addgene (https://www.addgene.org/) under reference numbers: #173505, #173665, #173666, #173667 for Nix-expressing piggyBac-based transgenesis plasmids and #173496 for piggyBac vector backbone. All plasmids and mosquito strains described in this paper are available upon request to EM. All data needed to evaluate the conclusions in the paper are present in the paper and/or the Supplementary Information.

Field-specific reporting

Please select the one below that is the best fit for your research. If you are not sure, read the appropriate sections before making your selection.

☒ Life sciences ☐ Behavioural & social sciences ☐ Ecological, evolutionary & environmental sciences

For a reference copy of the document with all sections, see nature.com/documents/nr-reporting-summary-flat.pdf

Life sciences study design

All studies must disclose on these points even when the disclosure is negative.

Sample size	No sample size pre-calculation was performed. Sample sizes were chosen as large as possible depending on mosquito availability and insectary maintenance capacity.
Data exclusions	No data were excluded except for extreme outliers within qPCR technical triplicates. When the standard deviation of a given triplicate was above 0.5Ct, only the duplicate ensuring minimum standard deviation was kept.
Replication	All experiments were repeated on 3 to 5 biological replicates of each line. Whenever possible, experiments were repeated on different transgenic lines. To ensure maximum reproducibility, no biological replicate was ever excluded.
Randomization	No randomization was involved. To avoid introducing bias through larval rearing conditions, mosquito larvae of the two lines to be compared were mixed in the same larval tray whenever possible, and sorted at the pupal stage based on their fluorescence.
Blinding	Blinding was not relevant in this study. All comparisons were based on objective or automated measurements.

Reporting for specific materials, systems and methods

We require information from authors about some types of materials, experimental systems and methods used in many studies. Here, indicate whether each material, system or method listed is relevant to your study. If you are not sure if a list item applies to your research, read the appropriate section before selecting a response.

Materials & experimental systems

n/a	Involved in the study	
☒	☐	Antibodies
☒	☐	Eukaryotic cell lines
☒	☐	Palaeontology and archaeology
☐	☒	Animals and other organisms
☒	☐	Human research participants
☒	☐	Clinical data
☒	☐	Dual use research of concern

Methods

n/a	Involved in the study	
☒	☐	ChIP-seq
☐	☒	Flow cytometry
☒	☐	MRI-based neuroimaging

Animals and other organisms

Policy information about studies involving animals; ARRIVE guidelines recommended for reporting animal research

Laboratory animals	Aedes albopictus mosquitoes from a domesticated local line were used for all experiments. Mice were used for mosquito blood-feeding.
Wild animals	The study did not involve wild animals.
Field-collected samples	The study did not involve samples collected from the field.
Ethics oversight	Mice use and rearing was approved by CREMEAS ethics comittee.

Note that full information on the approval of the study protocol must also be provided in the manuscript.

Flow Cytometry

Plots

Confirm that:

☒ The axis labels state the marker and fluorochrome used (e.g. CD4-FITC).

☒ The axis scales are clearly visible. Include numbers along axes only for bottom left plot of group (a 'group' is an analysis of identical markers).

☒ All plots are contour plots with outliers or pseudocolor plots.

☒ A numerical value for number of cells or percentage (with statistics) is provided.

Methodology

Sample preparation	COPAS is a device allowing large object flow cytometry that was used here for sorting mosquito first-instar larvae. Their fluorescence being endogenous, live 0.1 day old larvae were directly introduced in the sample tank without prior staining.
Instrument	COPAS SELECT (Union Biometrica) with 500µm flow cell.
Software	COPAS SELECT provided software: BIOSORT.
Cell population abundance	Several hundreds to several thousands of larvae were sorted at once. Abundance of the sorted population was measured by the software. Purity was ensured by proper gating on the extinction/time of flight graph and assessed post-sorting by visual screening under a fluorescence binocular microscope.
Gating strategy	Fluorescent versus non-fluorescence larvae were displayed on two distinct dot clusters. Following a first run of the sample in "Acquire" mode, "gate" and "sort" regions were defined so that all dots contained in the "sort" region were larvae of the desired fluorescence. When necessary, pilot sortings could be performed on a sub-sample and controlled visually under a fluorescence binocular microscope in order to refine the gating.

☒ Tick this box to confirm that a figure exemplifying the gating strategy is provided in the Supplementary Information.
